# Evaluating Telehealth Diagnostic Accuracy in Oral and Maxillofacial Diseases: A Comparative Study

**DOI:** 10.3390/jpm14121147

**Published:** 2024-12-10

**Authors:** Jeremy Lau, Agnieszka M. Frydrych, Richard Parsons, Ramesh Balasubramaniam, Omar Kujan

**Affiliations:** 1 UWA Dental School, The University of Western Australia, Nedlands, WA 6009, Australia; 21584322@student.uwa.edu.au (J.L.); agnieszka.frydrych@uwa.edu.au (A.M.F.); ramesh.balasubramaniam@uwa.edu.au (R.B.); 2 Medical School, Curtin University, Bentley, WA 6009, Australia; r.parsons@curtin.edu.au

**Keywords:** oral, maxillofacial, diseases, diagnosis, telehealth

## Abstract

**Objectives:** This study evaluated the accuracy of diagnosing oral and maxillofacial diseases using telehealth. **Methods:** We recruited 100 patients from the Oral Health Centre of Western Australia. They were either new patients or existing patients with a condition not previously diagnosed. The patients initially underwent a telehealth consultation without administrative or clinical staff assistance. On the same day, they also received a traditional in-office (face-to-face) consultation with an Oral Medicine registrar and/or consultant. **Results:** In this study, 70 patients were consulted via telehealth for a mucosal condition, while 30 were consulted for orofacial pain. When comparing telehealth diagnoses to in-person diagnoses, 65.7% of mucosal cases and 70% of orofacial pain cases had the same diagnosis. Furthermore, regarding the diagnoses within the chief complaint’s telehealth differential diagnosis list, 87.1% were similar in the mucosal cases, and 96.7% were similar in orofacial pain cases compared to the in-office consultation. **Conclusions:** Our study’s findings demonstrate that telehealth is less reliable than the gold standard in-person consultation for diagnosing oral diseases. However, it shows promise as an adjunctive service for screening, triaging, and monitoring patients. Further studies with guidelines for patients undergoing telehealth consultations to improve the diagnostic accuracy of oral and maxillofacial diseases are necessary.

## 1. Introduction

Telehealth involves remote diagnosis and treatment of patients through a telecommunications infrastructure [1]. In Australia, telehealth services are typically provided via phone, video call, emails or mobile phone applications. Telehealth allows access to healthcare that otherwise may not be available, especially for patients who are physically immobile or reside in a remote location. The COVID-19 pandemic led to unprecedented societal lockdowns across many countries around the globe. During this period, restrictions were imposed on healthcare provisions, and telehealth applications increased significantly [1,2].

Telehealth has the same Quadruple Aim of adequate healthcare as conventional consultations—that is, improving the patient’s experience of care, improving the health of populations, reducing the per capita cost of healthcare, and improving the experience of healthcare providers [3]. Telehealth has made access to healthcare more feasible, particularly specialist care for regional and remote residents. There is also evidence that telehealth may reduce healthcare costs, and in the short to medium term, it may reduce healthcare costs by approximately 50% [4].

Oral Medicine involves the diagnosis, prevention, and predominantly non-surgical management of medically related disorders affecting the oral and maxillofacial region. Typically, patients present with oral mucosal disease and orofacial pain [5]. The prevalence of oral mucosal lesions is 20–28% of the population [6,7], and regarding orofacial pain, there is a global prevalence of 32% and a mean annual incidence rate of approximately 2% [8]. These statistics imply a significant burden of disease.

A systematic review in 2020 reported a moderate-to-high agreement in diagnosing oral mucosal disease between in-person examinations and telehealth consultations [9]. Telehealth studies have varied methodologies, and it is interesting that photographs of oral disease utilised during telehealth consultations are obtained by oral healthcare professionals [9]. Regarding the diagnosis of temporomandibular disorders, telehealth appears to be effective [10]. Generally, the use of telehealth is well-accepted by patients and clinicians [11].

To date, no studies have investigated the diagnostic accuracy of telehealth in the full scope of Oral Medicine practice without any assistance from an oral health professional. It is hypothesised that telehealth is a relatively accurate form of consultation and is well-accepted by patients. This study assessed the accuracy of diagnosing and triaging oral and maxillofacial diseases using telehealth at a public Oral Medicine clinic. A secondary aim was to provide recommendations for its implementation in Oral Medicine practice in Australia.

## 2. Materials and Methods

### 2.1. Study Design and Participants

This was a prospective study following the STARD reporting guidelines [12], and was conducted between July 2023 and April 2024. This study received ethics approval from the Human Ethics Committee of the University of Western Australia (2023/ET000213). Convenience sampling (n = 100) was considered adequate based on previous studies investigating the accuracy and patients’ perceptions of telehealth services [11,13,14]. Patients with oral and maxillofacial diseases referred to the Oral Medicine Clinic at the Oral Health Centre of Western Australia (OHCWA) were reviewed for possible participation in this study. The inclusion criteria included new patients referred to the Oral Medicine Clinic or existing patients referred for a previously unseen condition. Exclusion criteria included follow-up consultations, patients under the age of 18 years, patients with a significant hearing impairment, or patients with poor vision or legally blind. Participants were invited, and a consecutive sampling method was utilised.

**Figure 1:**
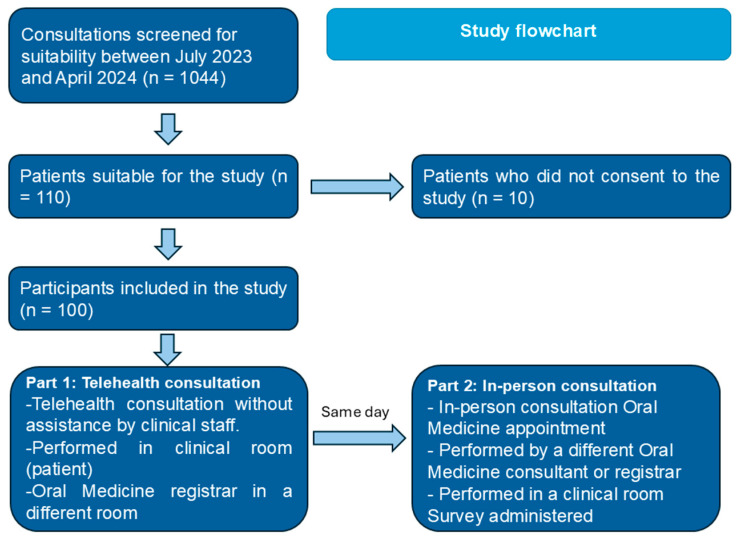
Flow diagram of the study design. The accuracy of diagnosis was compared between telehealth and in-person consultations. The telehealth and in-person consultations were completed on the same day.

Following informed consent, participants were enrolled in the study (Figure 1). Patients who participated had their initial Oral Medicine consultation performed using Coviu—a telehealth platform. The telehealth consultation consisted of a comprehensive medical history, a presenting complaint, and a visual examination through a webcam. This was performed by an Oral Medicine trainee (JL). Patients received no assistance from clinical staff but could receive assistance from an accompanying friend or family member where available. Patients attended an in-person Oral Medicine consultation with a different Oral Medicine trainee or Oral Medicine specialist consultant on the same day. The in-person consultation consisted of a comprehensive medical history and assessment of the presenting complaint and included a conventional oral examination with white light. Where applicable, photographs, biopsies or further investigations were organised on the same day or at a subsequent appointment.

An initial pilot study was conducted in July 2023, and two consenting patients participated. As a result of the pilot study, a telehealth oral examination protocol was established, along with the refinement of collected data. The data from the pilot study were used solely to devise the study’s workflow. The protocol for mucosal conditions followed protocols established by oral and maxillofacial surgeons [15]. Regarding orofacial pain conditions, the Diagnostic Criteria for Temporomandibular Disorders (DC/TMD) were followed for temporomandibular disorders [16], and the International Classification of Orofacial Pain (ICOP) was followed for other orofacial pain diagnoses [17]. Patients were routinely asked to self-palpate muscles of mastication when presenting with orofacial pain conditions. Patients were asked to palpate their muscles of mastication with approximately one kilogram of force as described in the DC/TMD protocol [16].

### 2.2. Questionnaire

Patients were also invited to complete a 10-question questionnaire (Supplementary Table S1). The questionnaire was based on a cross-sectional study investigating satisfaction with telehealth consultations in Oral Medicine in New Zealand [11]. The questions explored the participants’ experience of telehealth and in-person consultations and their demographics.

### 2.3. Referral Analysis

The quality of the referral received for each participant was analysed. It was recorded whether the referral had any radiographs or clinical photographs attached. Each referral was also scored based on its quality. This was modified based on a study evaluating a Brazilian Oral Medicine service [18]. Slightly different criteria were utilised for oral mucosal diseases compared to referrals for orofacial pain. (Supplementary Table S2) Each referral was scored out of 8, depending on whether specific criteria were fulfilled. The criteria were based on the lesion description, the history of the presenting condition, and a diagnostic hypothesis. The quality of referrals was classified as poor (0–2 points), regular (3–4 points), good (5–6 points), or excellent (7–8 points).

### 2.4. Statistical Analysis

Statistical analyses were performed using SAS version 9.4 (SAS Institute Inc., Cary, NC, USA, 2016). The diagnoses given over telehealth and in-person consultations were compared. The list of differential diagnoses from the telehealth consultation was also compared with the working diagnosis. The in-person diagnosis was considered the reference standard. After the computation of descriptive statistics, cross-tabulation with chi-squared and Fisher’s exact test were utilised to determine the accuracy of telehealth consultations. Statistical significance (*p* < 0.05) between groups, such as diagnoses of oral mucosal disease compared with orofacial pain conditions, was also examined. Other factors, including the quality of the referral, the provision of a clinical photograph or radiograph, and the risk of malignancy, were also cross-tabulated to analyse if they were associated with the accuracy of diagnosis. Furthermore, descriptive statistics were provided for the survey given to participants, and the data output was presented in a graphical format.

## 3. Results

### 3.1. General Characteristics, Patient Demographics, Categories of Seen Conditions, Referral Quality

One hundred subjects participated in this study (Table 1). Seventy participants presented for oral mucosal disease, and thirty presented for orofacial pain. The age range was between 19 and 91, with the mean age being 63. A third were males, and two-thirds were females. Seventy-nine participants spoke English as their primary language at home, whereas twenty-one patients spoke a language other than English as their primary language. There was also a range of distances travelled for patients to attend their in-person consultations. Thirty per cent of patients travelled at least an hour in each direction, ten per cent travelled 2 h each way, and one patient travelled 4 h to attend their appointment.

Oral mucosal disease and orofacial pain conditions were grouped according to their risk of malignancy from the in-person consultation. Of the 100 patients, there were 20 (20%) cases considered to be potentially oral malignant disorders (OPMD) or at risk of malignancy. The quality of referrals also ranged from poor to excellent. A total of 13% of the referrals were considered poor quality, 45% satisfactory, 37% good, and 5% excellent. Slightly over 50% of referrals had a radiograph attached, although most were screening orthopantograms. Furthermore, only 18% had a clinical photograph attached.

### 3.2. The Overall Accuracy of Telehealth

When combining all Oral Medicine-related conditions, the overall accuracy of telehealth was 67%. That is, 67% of cases had the same telehealth diagnosis as the in-person diagnosis. The accuracy of diagnosing oral mucosal disease was 65.71%, and orofacial pain was 70%, respectively (Table 2). Eighty-seven per cent of the in-person mucosal disease diagnoses were within the telehealth working differential diagnosis, and a corresponding accuracy of 96.67% for orofacial pain diagnoses. Despite the slightly higher accuracy of orofacial pain diagnosis, the difference was not considered statistically significant (*p*-value = 0.676).

Further analysis was also completed to determine if factors such as referral quality and the attachment of radiographs or photographs with the referral were associated with diagnostic accuracy (Table 3). For referrals that included a radiograph, the accuracy of diagnosis was 61.5%, and it was not statistically significantly different from those without a radiograph (*p* = 0.227). Referrals with a photo attached achieved a higher diagnostic accuracy of 72.2% compared to those without a photo (65.9%), but this difference was not statistically significant (*p* = 0.603). In addition, a poor-quality (53.8%) referral achieved a lower diagnostic accuracy compared to a satisfactory (75.6%), good, or excellent referral (61.9%), but these differences were not considered significantly significant (*p* = 0.223).

### 3.3. Diagnostic Accuracy Based on Risk of Malignancy

To address the risk of malignancy, categories of conditions were grouped into two groups: “at risk” and “not at risk”. OPMDs and malignant conditions were grouped into the “at risk” group (n = 20), and the other categories were grouped into the “not at risk” group (n = 80). When comparing telehealth to in-person diagnosis, the “at risk” group had a lower diagnosis accuracy than the “not at risk” group. The accuracy of diagnosis was 50% and 71.25%, respectively (*p* = 0.071). Similarly, the accuracy of the differential diagnosis list in “at risk” conditions followed a similar trend where the “at risk” group had a lower diagnostic accuracy of 80% (*p* = 0.096) (Table 4).

### 3.4. Survey Satisfaction Results

All participants who took part in the study completed the survey (Supplementary Table S1). The use of telehealth was well received by participants. Only 2% of participants were uncomfortable with telehealth use; similarly, only 1% were unsatisfied with the telehealth process. Only 4% of the participants reported that it was not worth their time, while 50% reported it was worth their time. When presented with either face-to-face or telehealth consultations, 64% of participants elected face-to-face, 19% chose telehealth, and 17% had no preference. Furthermore, 81% of participants would undergo a future telehealth consultation again.

**Figure 2:**
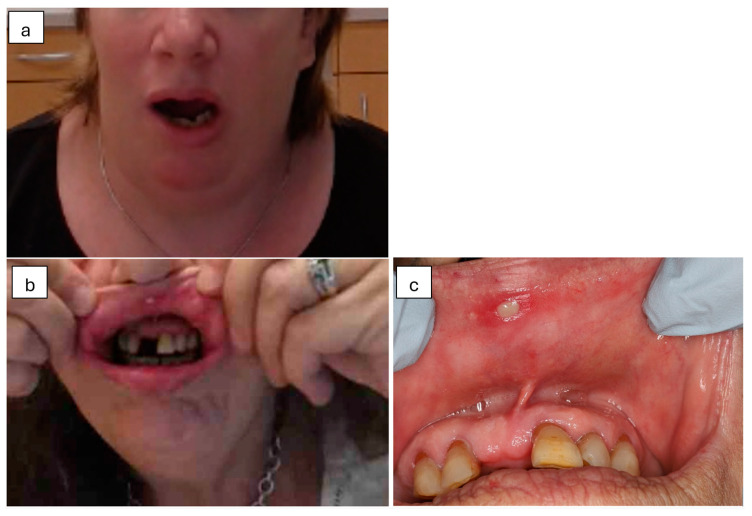
Clinical example of a telehealth consultation. (**a**) Deflection of the mandible to the left with limited mouth opening was diagnosed as left TMJ disc displacement without reduction and limited opening. (**b**) A solitary ulcer of the upper labial mucosa, diagnosed as an aphthous ulcer, observed during the telehealth consultation. (**c**) Clinical photograph of the same upper labial mucosa ulcer shown in 2b, captured during the in-person consultation.

Sixty-one per cent of participants considered the video quality very good and thirty-four per cent good. Despite the positive response of participants regarding the video quality, the Oral Medicine trainee performing the telehealth consultations found it challenging to visualise lesions in the posterior aspect of the mouth. The lighting and focus of the webcam were not considered optimal. An example of an orofacial pain patient during the telehealth consultation presented with limited mouth opening and left jaw deflection on mouth opening. The patient was assigned the diagnosis of left TMJ disc displacement without reduction and restricted mouth opening (Figure 2a). Another example of a solitary ulcerated lesion is noted (Figure 2b,c) on the patient’s anterior maxillary labial mucosa, approximately 4 × 4 mm in size with a surrounding erythematous border. With a clinical history of recurrent ulceration, the lack of parafunctional habits or trauma, and factoring in the medical history and clinical presentation, a telehealth working diagnosis of an aphthous ulcer was given.

## 4. Discussion

This study evaluates the accuracy of telehealth diagnosis compared with in-person consultations. Oral mucosal disease and orofacial pain had a diagnostic accuracy of 65.7% and 70%, respectively. The accuracy of the telehealth diagnosis in our study was similar to that of previous telehealth studies in Oral Medicine. A Malaysian study evaluating the accuracy of MeMoSa, a mobile phone application and telehealth tool, reported a detection accuracy of approximately 60% [19]. Another Brazilian study utilising email as a telehealth method compared findings from a conventional oral exam to digital photography. Two Oral Medicine consultants viewed the photographs remotely, and both specialists correctly diagnosed 60% of cases [20]. Most telehealth studies investigating the accuracy of orofacial pain have focused on TMD. A recent study reported extremely high levels of agreement between myalgia (91.5%) and arthralgia (86.3%). At the same time, other diagnoses such as degenerative joint disease and disc displacement with reduction and intermittent locking showed weak agreement [21]. To date, no meta-analysis on the accuracy of telehealth in Oral Medicine has been conducted. Differences in methodology and variations in telehealth applications utilised in other studies make it difficult to compare results. To emulate a realistic patient scenario, the participants in this study did not have assistance from other oral health professionals, which likely reduced the diagnostic accuracy.

This study reported high accuracy when comparing in-person diagnoses with telehealth differential diagnoses: 87.14% for oral mucosa disease and 96.67% for orofacial pain. This indicates that telehealth is a feasible adjunct to clinical practice. It has the potential for use in triaging and screening. The aims of screening and triaging are not diagnostic. Due to the significant burden of disease related to Oral Medicine conditions in Australia and limited access to Oral Medicine specialists, telehealth can be utilised to minimise the strain on resources.

Interestingly, this study reported a lower accuracy of diagnosis for OPMDs or conditions at risk of malignancy compared with other diagnoses. Although the difference was not statistically significant, it conveys the seriousness of misdiagnosing an OPMD or malignant condition. OPMDs can often be asymptomatic, and if the lesion is located in an area of the mouth, such as the floor of the mouth or the posterior aspect of the oral cavity, they may be difficult to visualise. In clinical practice, in addition to conventional oral examination and a biopsy, adjunctive tools can also be utilised to aid Oral Medicine specialists in diagnosing such conditions [22]. While telehealth can be used as an adjunct to current services, it cannot replace the gold standard of a conventional oral examination and biopsy, which requires an in-person appointment. It is acknowledged that there are no current criteria regarding the definition of “gold standard” [23]. A systemic review noted that a congenital oral examination had a pooled sensitivity of approximately 70% and a specificity of 85% for diagnosing OPMDs [24]. Ultimately, telehealth should not be relied on as the sole method to detect oral lesions or exclude OPMDs.

This study also explored potential associated factors such as the quality of referrals. In Australia, new Oral Medicine consultations typically require a referral. A study in Italy investigating referral patterns in Oral Medicine noted that only 28.5% of patients had correct provisional diagnoses from their referrer [25]. In addition, the quality of referrals in Oral Medicine can be variable. A Brazilian study analysing 500 Oral Medicine referrals reported that approximately 40% were of poor quality, 50% were of satisfactory quality, and the remainder were good or excellent [18]. Although the difference in referral quality was not statistically significant in our study, referrals of poor quality had the lowest telehealth diagnostic accuracy of 53.85%, while regular and good/excellent referrals had 75.56% and 61.90% accuracy, respectively. The link between poor-quality referrals and lower telehealth diagnostic accuracy likely relates to the limitations of telehealth. Visualisation of lesions in the posterior aspect of the oral cavity, palatal or lingual mucosa, and floor of the mouth can be difficult via telehealth. The risk of misdiagnosis is significant with poorly described oral lesions or lesions not described at all in the referral. Furthermore, a cross-sectional study reported that the diagnostic accuracy of oral and maxillofacial lesions made by referrers was approximately 30%. A total of 58% did not have a determined diagnosis in their referral, and 12% had an incorrect diagnosis [26]. Although referral quality was not a statistically significant factor in our study, the clinical implications of a poor-quality referral may still be detrimental to overall outcomes for patients with telehealth. This is of particular concern when coupling the diagnostic accuracy of a poor-quality referral in our study with the percentage of poor-quality referrals seen in Oral Medicine [18].

The diagnosis of oral and maxillofacial diseases is not based on visualisation alone. A consultation involves gathering information about the patient’s chief complaint and a thorough medical history [27]. A satisfactory history can be obtained through telehealth or a traditional in-person consultation. Telehealth can be used to build rapport between clinicians and patients, and consequently, history-taking via telehealth should not be compromised [28]. Taking a good history is a significant factor influencing the diagnostic accuracy of Oral Medicine consultations. With telehealth consultations, a good history is likely a significant advantage over the common scenario of a clinical photograph alone with little or no history attached to the referral.

This study attempted to emulate a realistic telehealth environment without assistance from any oral health professionals. To have an ideal representation of this scenario, patients should perform telehealth consultations from their homes. Ultimately, we decided against this notion, as oral lesions not seen on the same day might present differently or resolve with or without treatment. In addition, the visualisation of certain aspects of the mouth is complex with telehealth. Adequate lighting and focusing on an oral lesion with a webcam is considered challenging. The diagnostic accuracy of telehealth can be improved with assistance. This was reported in a Brazilian prospective cross-sectional study where a general dentist performed face-to-face consultations before sending images and information to a remote specialist. The study achieved a 100% sensitivity and 97.5% specificity [29]. Furthermore, a study utilising smartphone video calls and images obtained by a general dentist reported over 90% concordance with telehealth [30]. It is recognised that having a dental or medical professional site to assist in the telehealth consultation provides a higher accuracy of diagnosis compared with our study. In remote areas where an Oral Medicine specialist is physically unable to attend, but general dentistry is available, this is the recommended method of performing telehealth.

The examination of orofacial pain cases, particularly TMD, is considered technique-sensitive. Specific muscles of mastication, such as the temporalis and masseter, should be palpated with 1 kg of force, as outlined in the DC/TMD [16]. The pain experienced should also be reproducible during an examination. In reality, remote examination, particularly palpation by the patient, is considered limited, and the force applied during palpation may not be accurately performed. In spite of these limitations, orofacial pain cases achieved a diagnostic accuracy of 70%.

A limitation of this study was that the quality of clinical photographs or video calls was not evaluated. In selected cases, as depicted in Figure 2a,b, screenshot captures were taken during video conferencing to depict certain clinical presentations. Where applicable, clinical photographs were taken during the in-person examination, as per standard Oral Medicine operating procedures. Previous studies have analysed the quality of photographs during remote oral examinations [31,32]. Photographic quality was rated as either good or poor based on criteria such as framing, focus, and exposure. Over 90% of photographs were considered good quality and were taken by dental or medical professionals present with the patient [31].

Furthermore, this study also examined patient satisfaction with telehealth consultations. Overall, there was high acceptance of telehealth, and 81% of patients would undergo a telehealth consultation again. A previous New Zealand study reported that 95% of patients were comfortable with telehealth [11]. Similarly, our study found that 95% of patients were either satisfied or very satisfied with telehealth (Supplementary Figure S1). Given the high acceptance rate of telehealth in Oral Medicine, there is certainly a role for its use. Notably, 10% of patients in this study travelled over 2 h each way to reach their appointment. Accounting for loss of income, time, and costs associated with travelling, telehealth has cost-effective advantages. Our study highlights the transformative potential of telehealth in diagnosing and managing oral lesions, emphasising its significant contributions to sustainability and healthcare accessibility. By facilitating remote consultations, telehealth minimises the need for patients to undertake long-distance travel—a crucial benefit in geographically expansive countries like Australia, where many people live in rural or remote areas. This approach not only reduces the environmental impact associated with travel but also streamlines healthcare delivery by prioritising in-person visits for urgent or complex cases, thereby optimising resource allocation. Additionally, telehealth offers substantial cost-effective advantages by mitigating the financial and personal burdens associated with travel, including loss of income, time, and related expenses.

While our study does not recommend telehealth replacing in-person consultations, telehealth can be utilised as an adjunctive service in clinical practice. Telehealth consultations have a high accuracy rate when comparing in-person and telehealth differential diagnoses. This suggests that telehealth may be utilised as screening or triaging, particularly in presentations of orofacial pain. However, caution is recommended with oral mucosal diseases considered “at risk”, such as malignant conditions and OPMDs. Such means have the potential to better distribute and prioritise in-person Oral Medicine consultations. Furthermore, our study recommends that telehealth be used for follow-up appointments, primarily to discuss clinical results. However, it should not be solely relied upon to monitor the progression of oral mucosal diseases and malignant transformation.

### Recommendations for Implementation of Telehealth in Oral Medicine

Recommendation 1: Telehealth can be utilised as an adjunctive service in clinical practice. It is overall less reliable than the gold-standard in-person consultation for diagnosing oral mucosal diseases and orofacial pain conditions.
Recommendation 2: Telehealth can be utilised as a screening or triaging service in Oral Medicine.
Recommendation 3: Telehealth should not be utilised to replace in-person examinations when monitoring OPMDs.
Recommendation 4: Referrals to Oral Medicine telehealth appointments should be of at least satisfactory quality. A clinical photograph for mucosal conditions is recommended in the referral.
Recommendation 5: Where possible, video calling is the suggested method of telehealth communication.
Recommendation 6: Given that Oral Medicine is a specialist service, a dental or medical health professional in the room assisting the patient is likely to increase the diagnostic accuracy.

## 5. Conclusions

This study provides evidence of high accuracy when comparing the current standard of in-person diagnosis to telehealth differential diagnosis. Although the diagnostic accuracy falls short of the gold standard, telehealth can be feasibly employed for screening, triaging, and follow-up consultations. Given Australia’s burden of oral diseases and the geographic vastness of the country, coupled with the fact almost all Oral Medicine specialists practice in capital cities, telehealth is a useful adjunctive service. It is anticipated that the utilisation of telehealth within Oral Medicine will continue to increase in the foreseeable future.

## Figures and Tables

**Table 1:** General characteristics, including patient demographics, consultation type, condition category, referral quality, and radiograph or photograph attached to the referral.

General Characteristics	Number (%)
**Gender**	
Male	33 (33%)
Female	67 (67%)
**Age**	
<30	3 (3%)
30–39 yo	8 (8%)
40–49 yo	8 (8%)
50–49 yo	15 (15%)
60–69 yo	22 (22%)
70–79 yo	36 (36%)
≥80	8 (8%)
**Language spoken at home**	
English	79 (79%)
Other	21 (21%)
**Time taken to travel (each way)**	
Under 30 min	18 (18%)
30–60 min	51 (51%)
1–2 h	21 (21%)
2–4 h	9 (9%)
4+ h	1 (1%)
**Consultation type**	
Oral mucosal disease	70 (70%)
Orofacial pain	30 (30%)
**Category of conditions**	
Clinically normal	8 (8%)
Benign	46 (46%)
Temporomandibular disorders	16 (16%)
Neuropathic pain	4 (4%)
Movement disorder	1 (1%)
Neurovascular pain	1 (1%)
Idiopathic/nociplastic pain	4 (4%)
Oral Potentially Malignant Disorder (OPMD)	20 (20%)
Malignant	0 (0%)
**Referral quality**	
0–2 (poor quality)	13 (13%)
3–4 (satisfactory quality)	45 (45%)
5–6 (good quality)	37 (37%)
7–8 (excellent quality)	5 (5%)
**Radiograph accompanied referral**	
Yes	52 (52%)
No	48 (48%)
**Photograph accompanied referral**	
Yes	18 (18%)
No	82 (82%)

**Table 2:** Diagnostic accuracy of oral mucosal disease and orofacial pain consultations, and accuracy of diagnosis within the working differential diagnosis.

Appointment Type	Diagnostic Accuracy (Working Diagnosis) *			Diagnosis Accuracy (Working Differential Diagnoses List) **		
	Number of Actual Correct Diagnoses/Total Number of Cases	Accuracy Rate (%)	*p*-Value	Number of Correct Differential Diagnoses/Total Number of Cases	Accuracy Rate (%)	*p*-Value
Oral mucosal disease	46/70	65.7	0.6762	61/70	87.1	0.1457
Orofacial pain	21/30	70		29/30	97	

* Comparing the working diagnosis of telehealth to in-person. ** Comparing the telehealth differential diagnosis to the in-person working diagnosis. Clinically significant *p*-value: ≤0.05. The *p*-value compares the accuracy rate of mucosal versus OFP.

**Table 3:** Diagnostic accuracy based on the quality of referrals and the inclusion of a radiograph or photograph. Good and excellent referrals are combined into a single category.

Referral Quality	Diagnostic Accuracy (Working Diagnosis) *		
	Number of Actual Correct Diagnoses/Total Number of Cases	Accuracy Rate (%)	*p*-Value
**Poor**	7/13	53.85	0.2231
**Satisfactory**	34/45	75.56	
**Good/Excellent**	26/42	61.90	
**Radiograph in referral**			
**Yes**	32/52	61.54	0.2267
**No**	35/48	72.92	
**Photograph in referral**			
**Yes**	13/18	72.22	0.6028
**No**	54/82	65.85	

* Comparing the working diagnosis of telehealth to in-person. Clinically significant *p*-value: ≤0.05. The *p*-value compares the diagnostic accuracy across differing referral qualities, ranging from poor to excellent. It also examines whether the inclusion of a radiograph or photograph in the referral makes a difference in the diagnostic accuracy of telehealth.

**Table 4:** Diagnostic accuracy based on risk of malignancy. Appointment categories were split into 2 groups based on malignant potential. Group one includes OPMDs and malignant conditions, while group two includes all other diagnoses (clinically normal, benign, TMD, neuropathic pain, movement disorders, neurovascular pain, idiopathic pain).

Category of Appointments Based on Risk of Malignancy	Diagnostic Accuracy (Working Diagnosis) *			Diagnosis Accuracy (Differential List) **		
	Number of Actual Correct Diagnoses/Total Number of Cases	Accuracy Rate (%)	*p*-Value	Number of Correct Differential Diagnoses/Total Number of Cases	Accuracy Rate (%)	*p*-Value
Group one—not at risk	57/80	71.25	0.0707	74/80	92.50	0.0956
Group two—at risk	16/20	50		16/20	80	

* Comparing the working diagnosis of telehealth to in-person. ** Comparing the telehealth differential diagnosis to the in-person working diagnosis. *p*-value—clinically significant *p*-value: ≥0.05. The *p*-value compares the accuracy rate between group one and group two.

## Data Availability

The data presented in this study are available on request from the corresponding author.

## Supplementary Materials

The following supporting information can be downloaded at https://www.mdpi.com/article/10.3390/jpm14121147/s1, Figure S1: Graphical output of participants’ responses in patient satisfaction surveys; Table S1: Survey given to patients to assess their satisfaction with telehealth; Table S2: Criteria utilised in grading the quality of referral.
