# Supplementary material for: Evaluating Telehealth Diagnostic Accuracy in Oral and Maxillofacial Diseases: A Comparative Study
Source: J Pers Med. 2024 Dec 10;14(12):1147. doi: 10.3390/jpm14121147 (PMC11677737; doi:10.3390/jpm14121147)
Supplement: Supplementary file 1 [file jpm-14-01147-s001.zip › jpm-3341585-supplementary.pdf]

Supplementary Materials for review

Supplementary Table S1: survey given to patients to assess their satisfaction with telehealth

Tele Oral Medicine Patient Satisfaction Survey

1. How comfortable did you feel?

Not comfortable at all ○ — Uncomfortable ○ — Neutral ○ — Comfortable ○ — Very comfortable ○

2. Were you satisfied with the process of a Tele Oral Medicine appointment?

Not satisfied at all ○ — Unsatisfied ○ — Neutral ○ — Satisfied ○ — Very satisfied ○

3. The use of Tele Oral Medicine is worth my time?

Not worth my time at all ○ — Not worth my time ○ — Neutral ○ — Worth my time ○ — Definitely worth my time ○

4. How would you rate the video conference quality?

Very poor ○ — Poor ○ — Neutral ○ — Good ○ — Very good ○

5. I prefer the use of Tele Oral Medicine over face to face visits?

Prefer face to face ☐
Prefer Tele Oral Medicine ☐

6. I find Tele Oral Medicine a feasible (reasonable and practical) service?

Not feasible at all ○ — Unfeasible ○ — Neutral ○ — Feasible ○ — Very feasible ○

7. Would you do a Tele Oral Medicine appointment again in the future?

Yes ☐
No ☐

Patient demographics

8. Gender

Male ☐
Female ☐
Other ☐

9. How much time did you spend travelling (one way) to the Oral Medicine Clinic?

Under 30 minutes ☐
30-60 minutes ☐
1-2 hours ☐
2-4 hours ☐
4+ hours ☐

10. What is your language spoken at home?

____

Supplementary Table S2: Criteria utilised in grading the quality of referral

	**Oral Mucosal Disease**	**Orofacial Pain**
1	Primary morphology of the lesion	Onset
2	Location	Location
3	Size	Duration or temporal behaviour: frequency/periods of remission
4	Limits	Quality of pain/intensity
5	Consistency	Modulating factors (aggravating/relieving)

6	History of presenting illness	History of presenting illness
7	Evolution history	Evolution history
8	Diagnostic hypothesis	Diagnostic hypothesis

Supplementary Figure S1: graphical output of participants' responses in patient satisfaction surveys

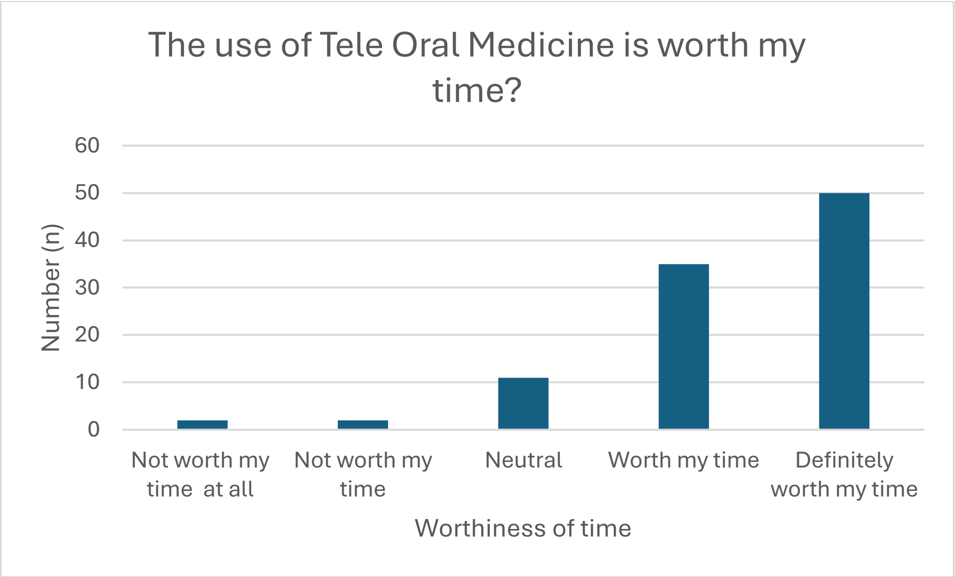

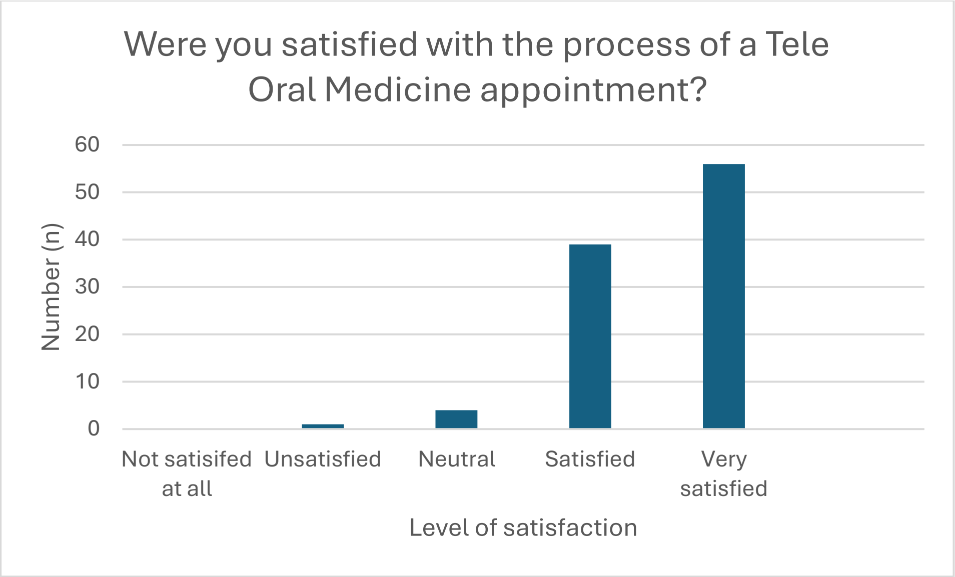

Were you satisfied with the process of a Tele Oral Medicine appointment?
Number (n)
60
50
40
30
20
10
0
Not satisifed at all
Unsatisfied
Neutral
Satisfied
Very satisfied
Level of satisfaction

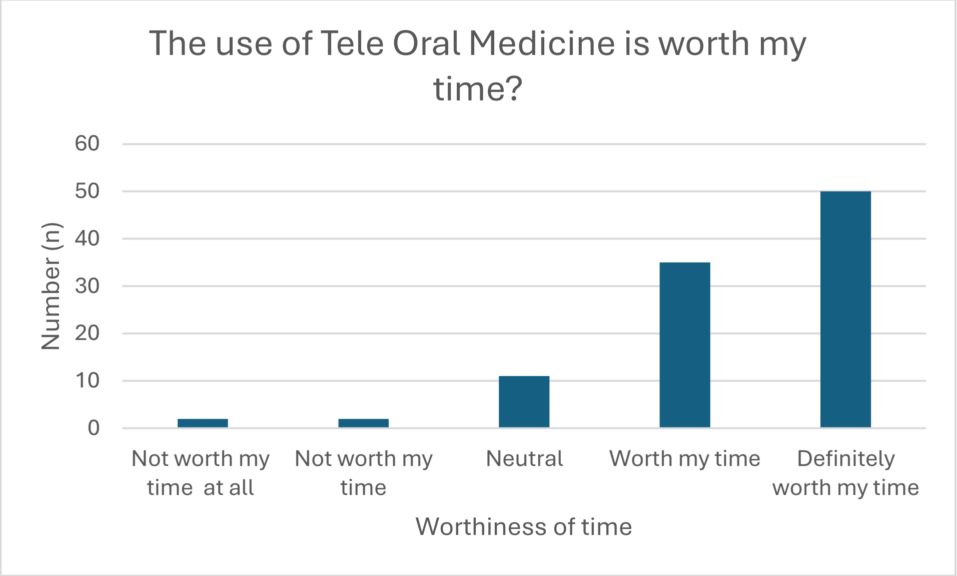

The use of Tele Oral Medicine is worth my time?
Number (n)
60
50
40
30
20
10
0
Not worth my time at all
Not worth my time
Neutral
Worth my time
Definitely worth my time
Worthiness of time

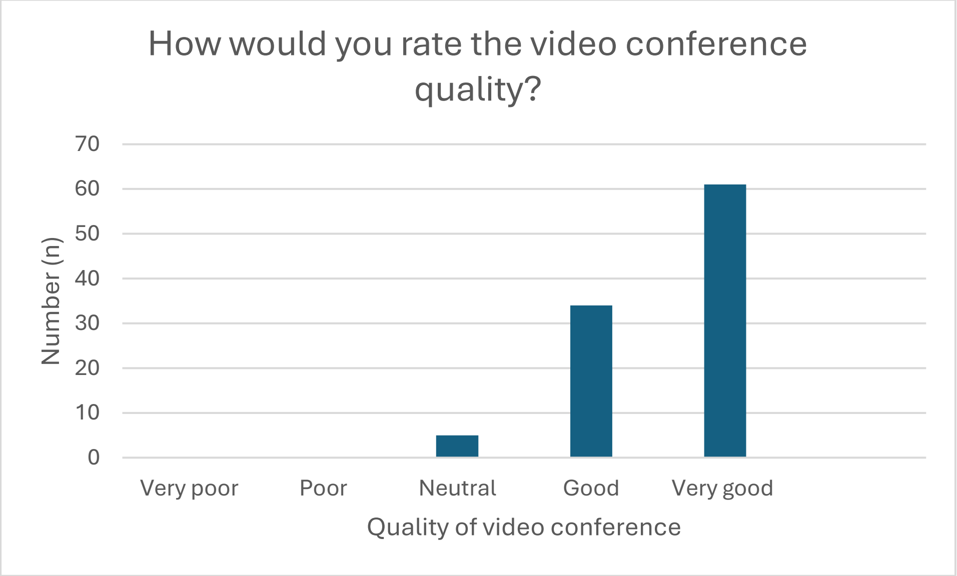

How would you rate the video conference quality?
Number (n)
70
60
50
40
30
20
10
0
Very poor
Poor
Neutral
Good
Very good
Quality of video conference

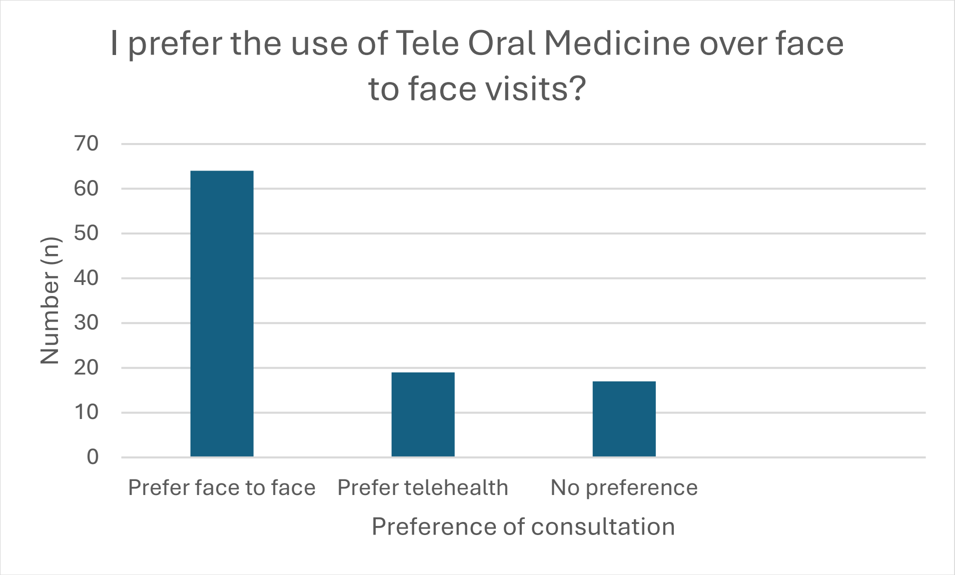
I prefer the use of Tele Oral Medicine over face to face visits?
70
60
50
40
30
20
10
0
Number (n)
Prefer face to face
Prefer telehealth
No preference
Preference of consultation

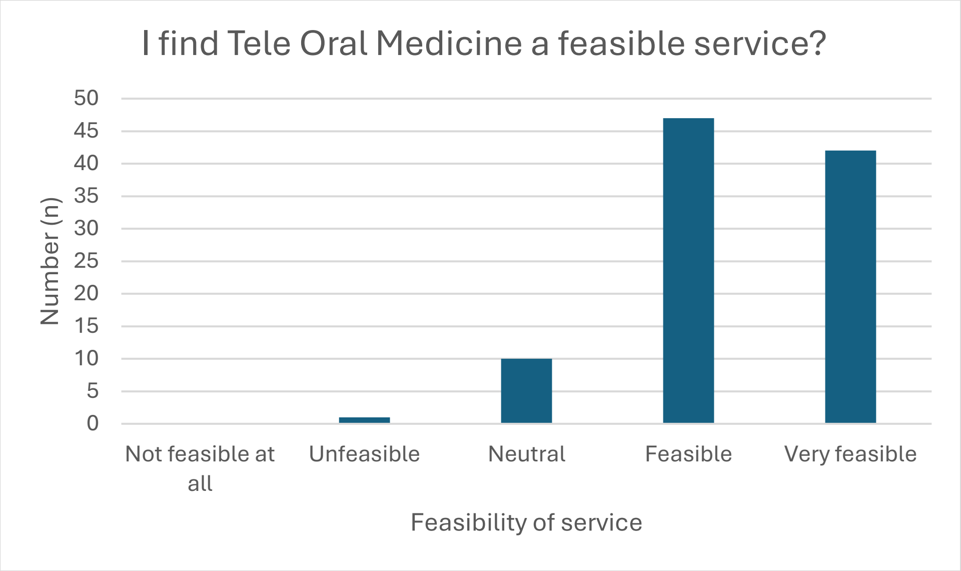
I find Tele Oral Medicine a feasible service?
50
45
40
35
30
25
20
15
10
5
0
Number (n)
Not feasible at all
Unfeasible
Neutral
Feasible
Very feasible
Feasibility of service

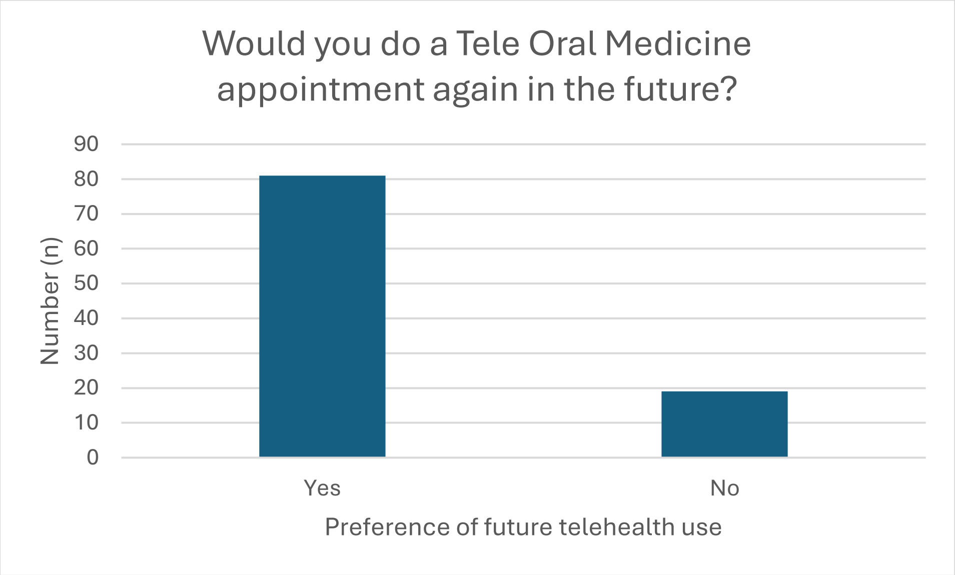
Would you do a Tele Oral Medicine appointment again in the future?
90
80
70
60
50
40
30
20
10
0
Number (n)
Yes
No
Preference of future telehealth use
